# Supplementary figures and images for: Construction and validation of prognosis and treatment outcome models based on plasma membrane tension characteristics in bladder cancer
Source: PeerJ. 2025 Jan 6;13:e18816. doi: 10.7717/peerj.18816 (PMC11716045; doi:10.7717/peerj.18816)

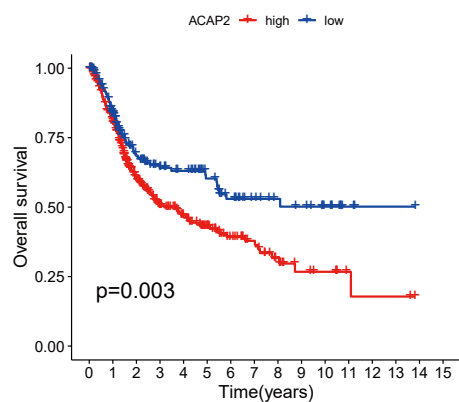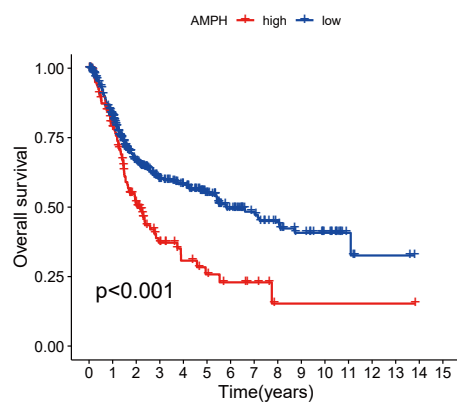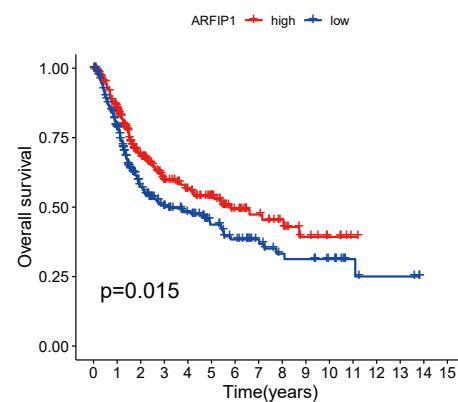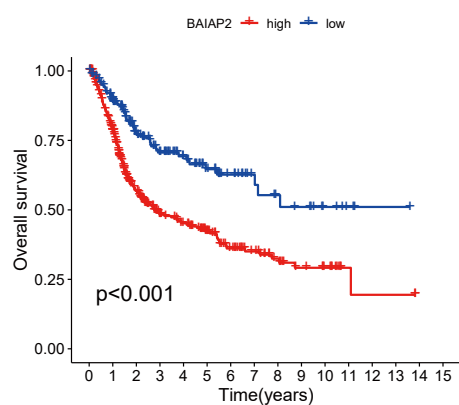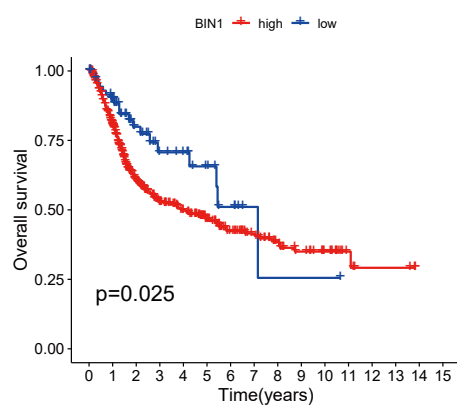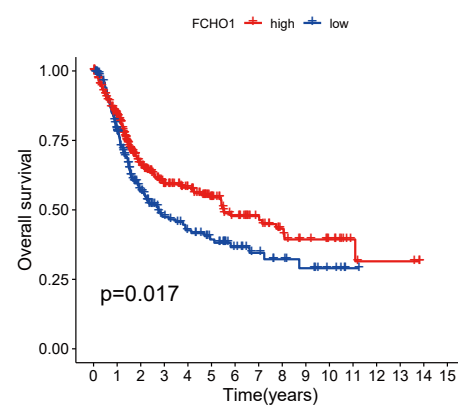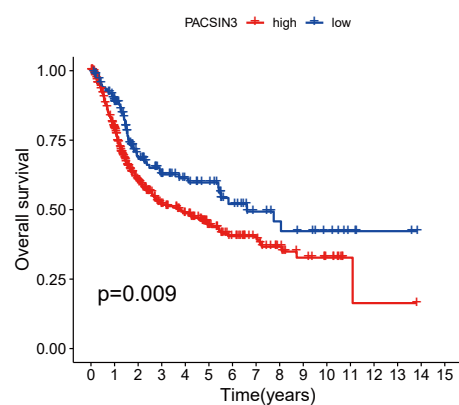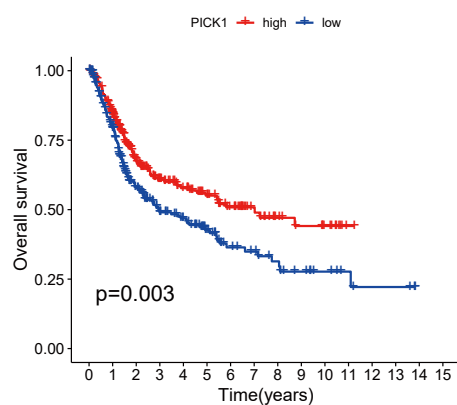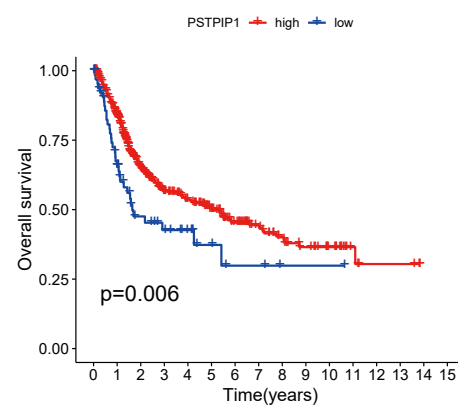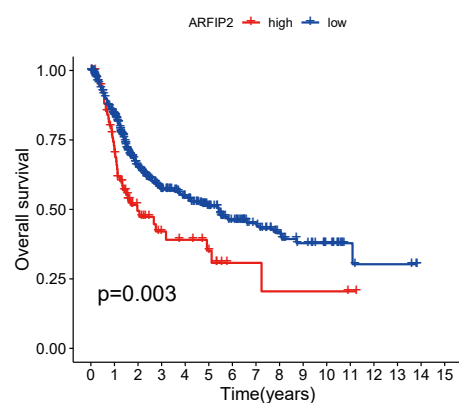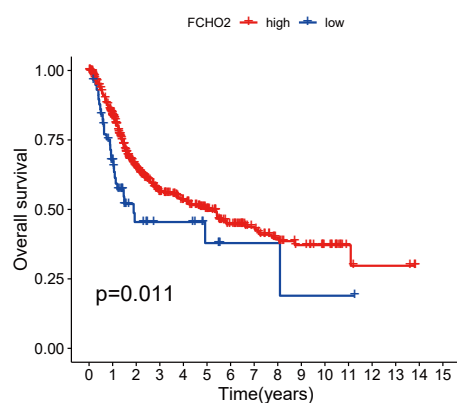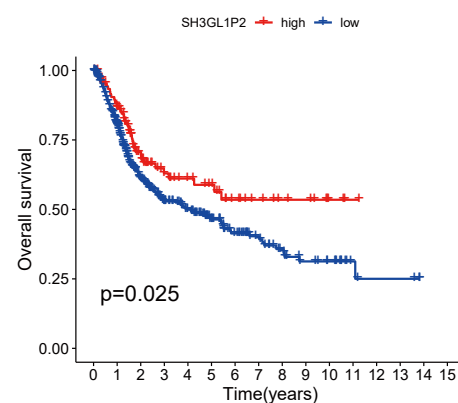

Supplement: Figure S1 [file peerj-13-18816-s001.pdf]

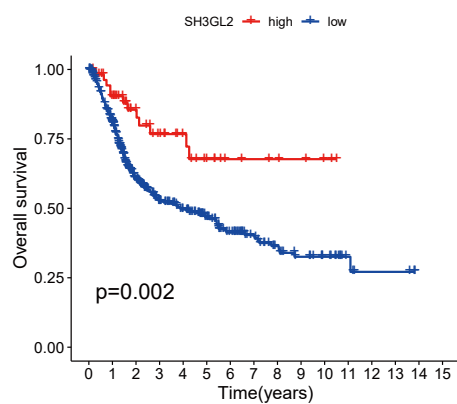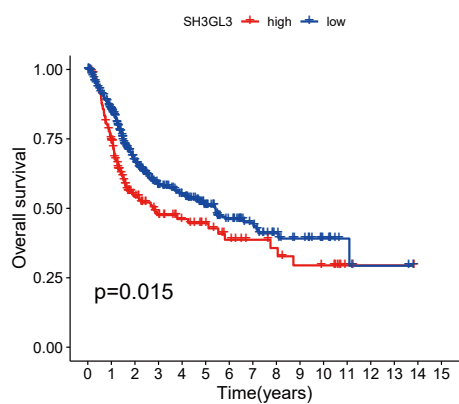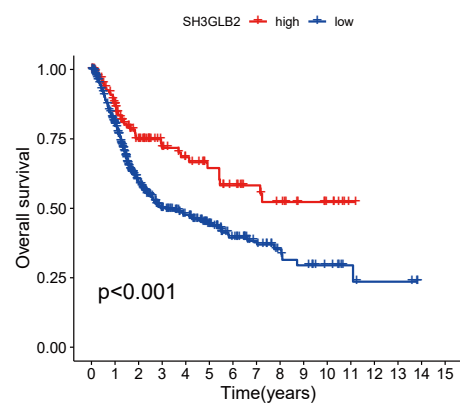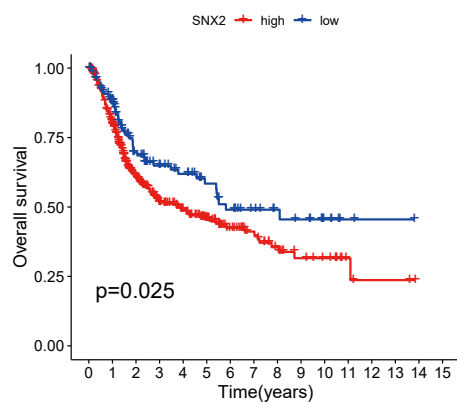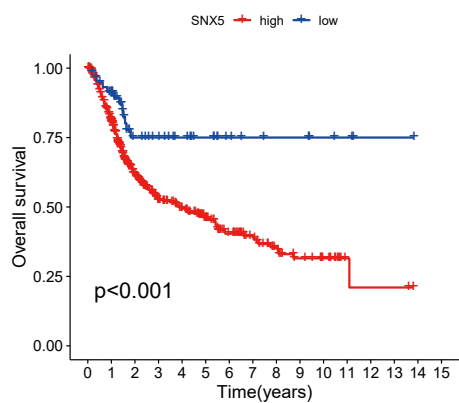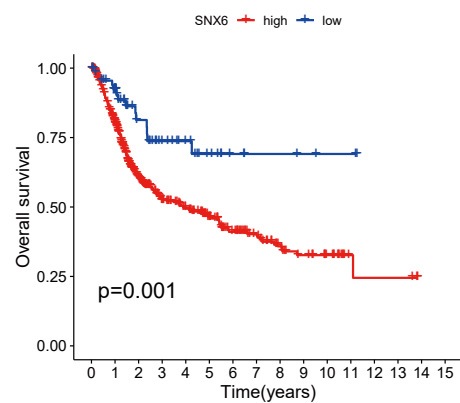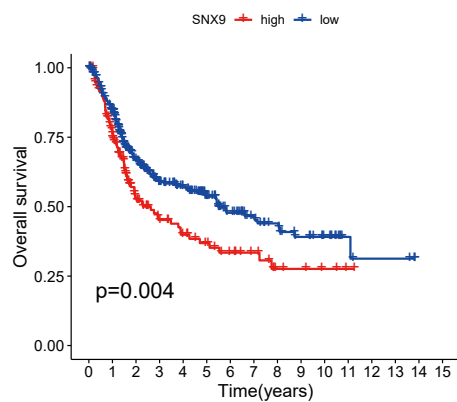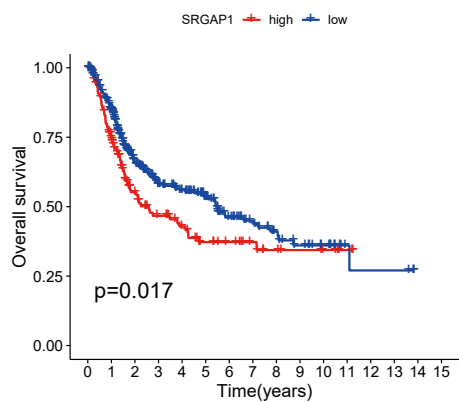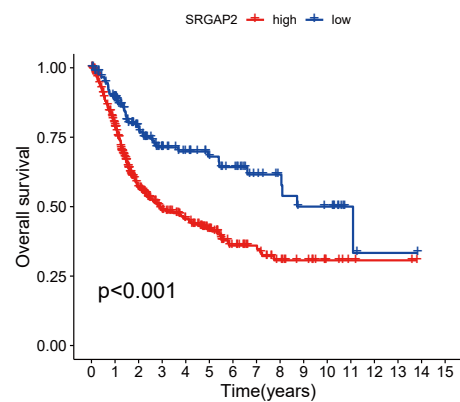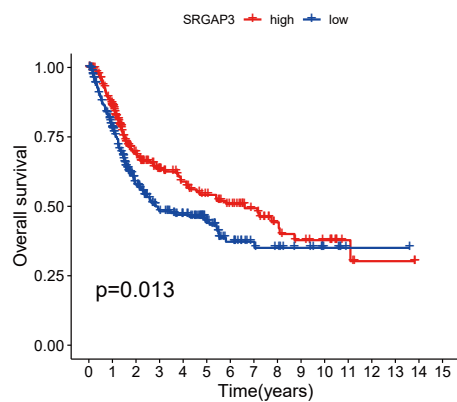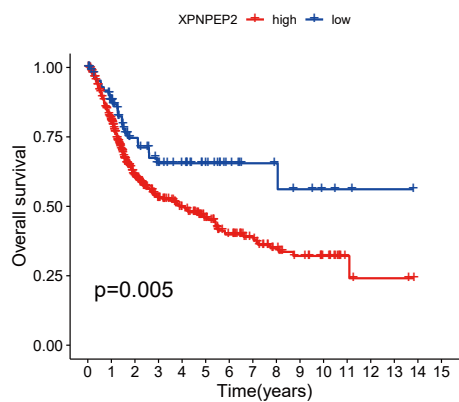

Supplement: Figure S2 [file peerj-13-18816-s002.pdf]

**A**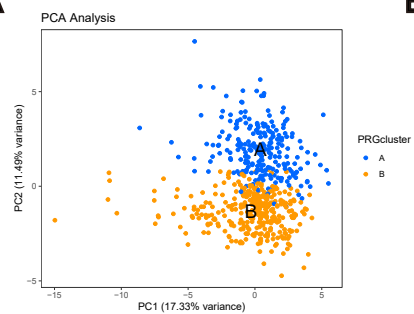**B**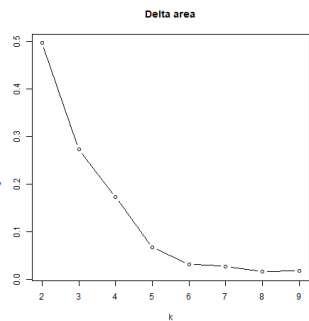**C**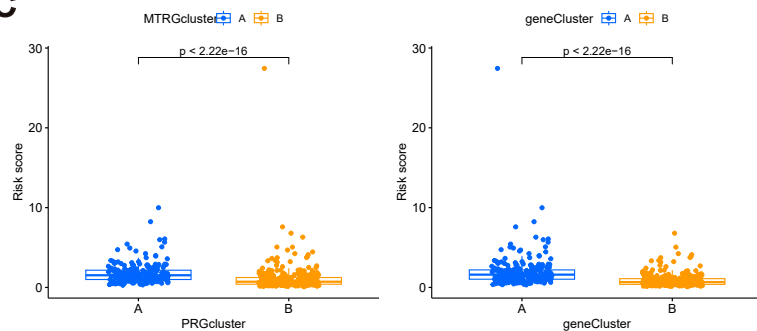**D**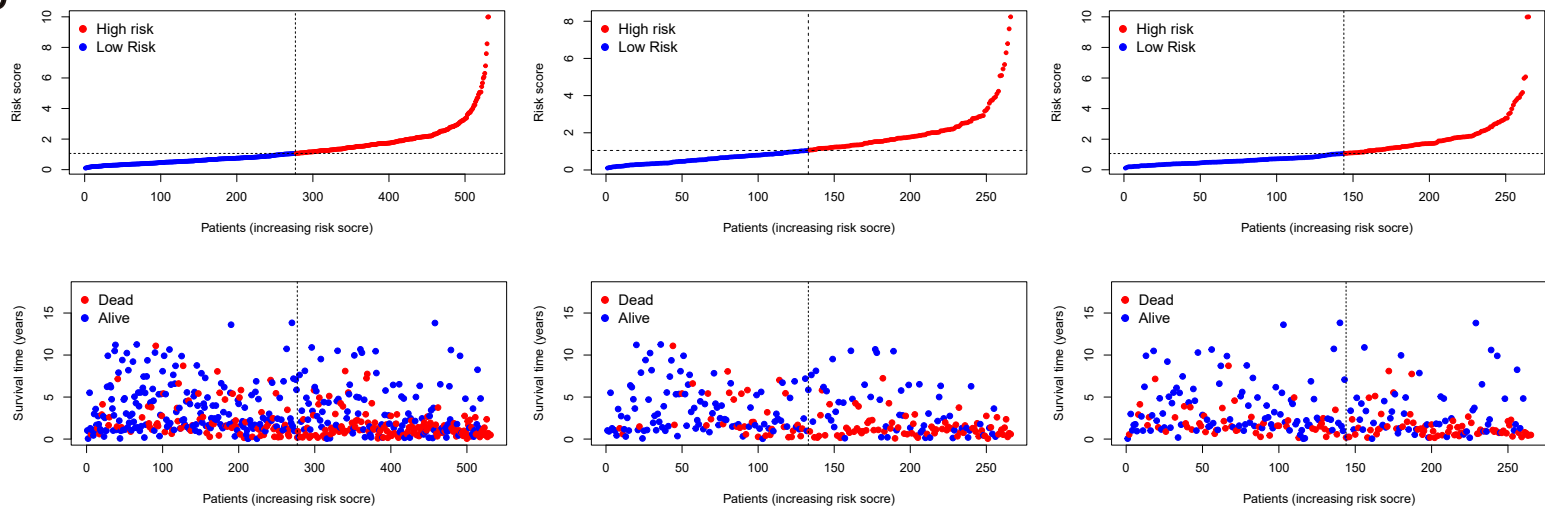**E**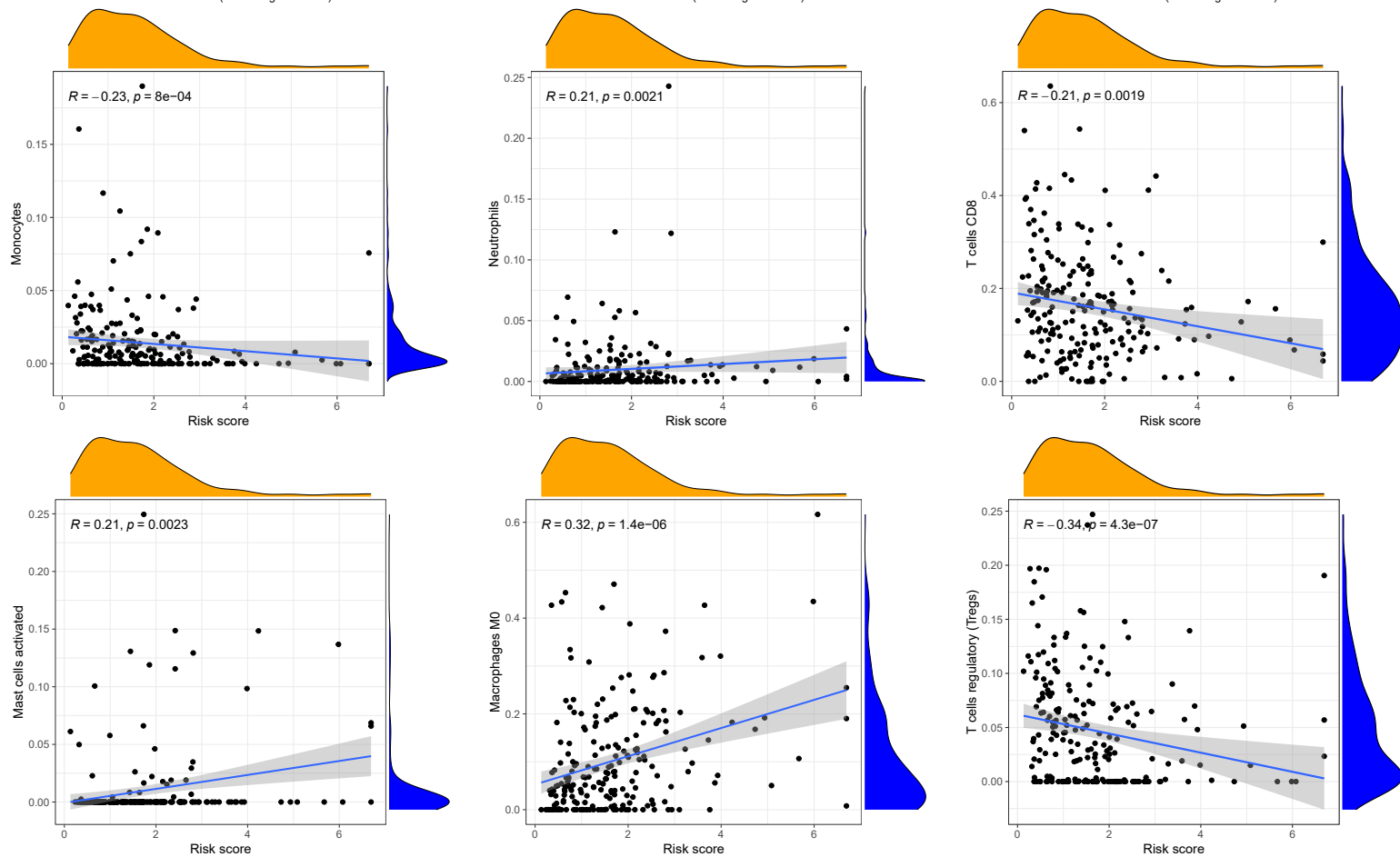

Supplement: Figure S3 — (A) Principal component analysis (PCA) between different molecular subtypes. (B) Consensus cluster analysis of prognostic DEGs. (C) Risk score differences between molecular and genetic subtypes. (D) Scatter plots of risk score distributions and survival rates for the pooled, training, and test sets. (E) Correlation between MTRG risk score and immune cells. [file peerj-13-18816-s003.pdf]

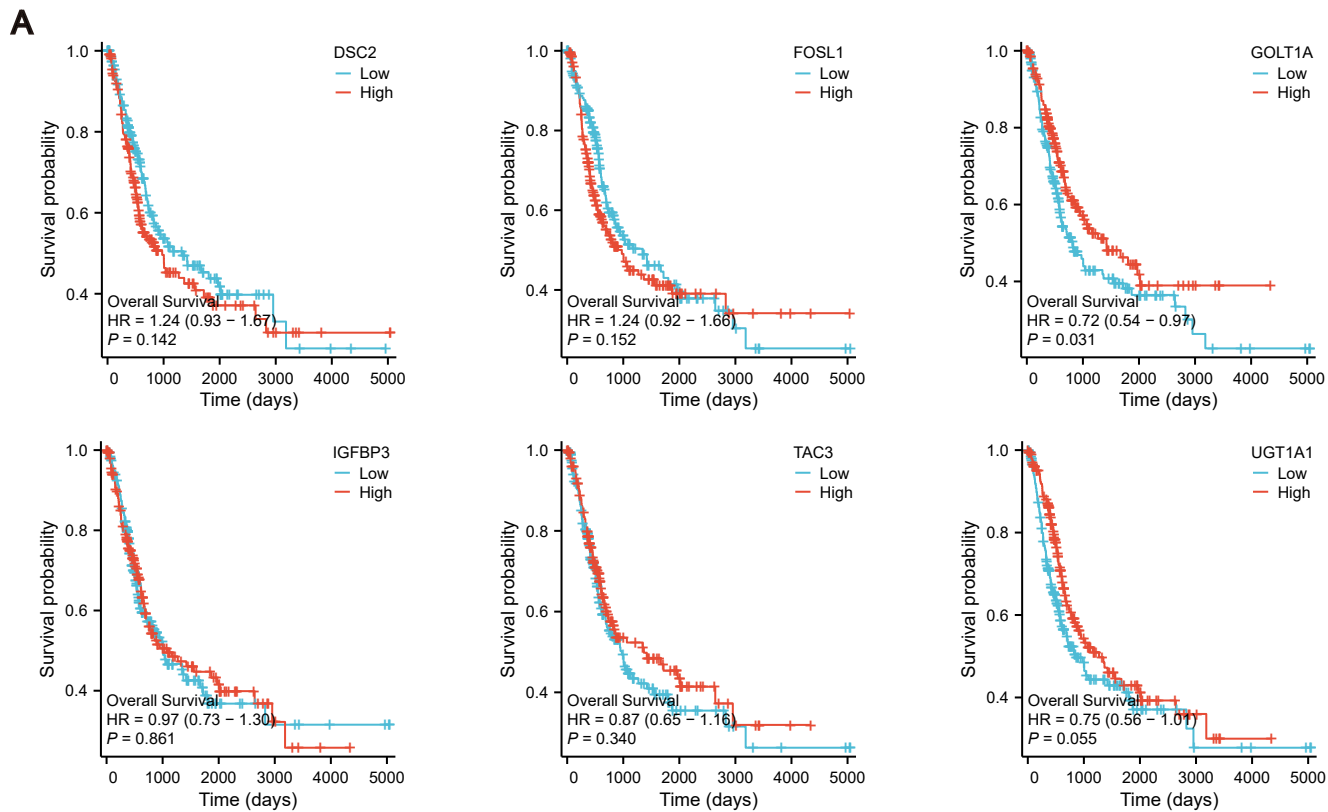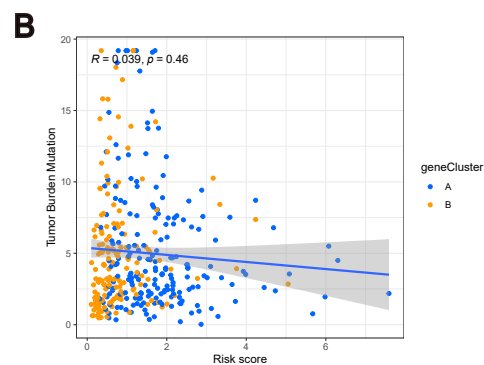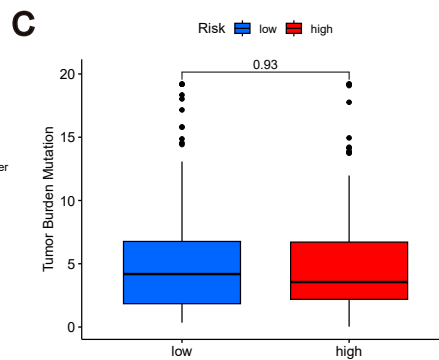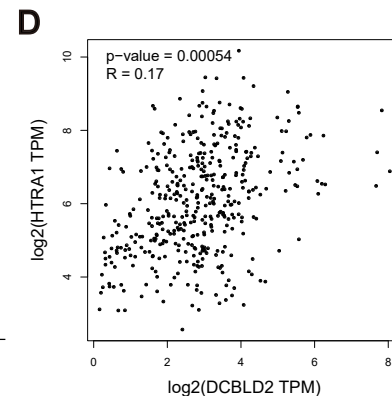

Supplement: Figure S4 [file peerj-13-18816-s004.pdf]
